# Extracellular self-RNA: A danger elicitor in pepper induces immunity against bacterial and viral pathogens in the field

**DOI:** 10.3389/fpls.2022.864086

**Published:** 2022-09-26

**Authors:** Doyeon Kim, Myoungjoo Riu, Sang-Keun Oh, Choong-Min Ryu

**Affiliations:** ^1^Molecular Phytobacteriology Laboratory, Infectious Disease Research Center, KRIBB, Daejeon, South Korea; ^2^Department of Biosystems and Bioengineering, KRIBB School of Biotechnology, University of Science and Technology, Daejeon, South Korea; ^3^Department of Applied Biology, College of Agriculture & Life Sciences, Chungnam National University, Daejeon, South Korea

**Keywords:** DAMP, trade-off, self-eRNA, plant immunity, pepper

## Abstract

Plants and animals serve as hosts for microbes. To protect themselves from microbe-induced damage, plants and animals need to differentiate self-molecules/signals from non-self, microbe-derived molecules. Damage-associated molecular patterns (DAMPs) are danger signals released from the damaged host tissue or present on the surface of stressed cells. Although a self-extracellular DNA has previously been shown to act as a DAMP in different plant species, the existence of a self-extracellular RNA (eRNA) as a danger signal in plants remains unknown. Here, we firstly evaluated the ability of a pepper self-eRNA to activate immunity against viral and bacterial pathogens under field conditions. Pepper leaves pre-infiltrated with self-eRNA exhibited reduced titer of the naturally occurring *Tomato spotted wilt virus* and diminished symptoms of *Xanthomonas axonopodis* pv. *vesicatoria* infection through eliciting defense priming of abscisic acid signaling. At the end of the growing season at 90 days after transplanting, pepper plants treated with self- and non-self-eRNAs showed no difference in fruit yield. Taken together, our discovery demonstrated that self-eRNA can successfully activate plant systemic immunity without any growth penalty, indicating its potential as a novel disease management agent against a broad range of pathogenic microbes.

## Introduction

Plants and animals serve as hosts for pathogenic microbes and suffer microbe-induced damage (Heil and Vega-Muñoz, 2019). In animals, the perception of and response to host-derived (self) and pathogen-derived (non-self) molecules have been investigated for a long time (Schlee and Hartmann, 2016; Barbero et al., 2021). By contrast, in plants, the perception of self- and non-self-signals and response to damage-associated molecular patterns (DAMPs) have not received sufficient attention (Bhat and Ryu, 2016; Heil and Vega-Muñoz, 2019). In 2015, Mazzoleni and colleagues were the first to report the autotoxicity of self-extracellular DNA (self-eDNA) as a mechanism of negative plant–soil feedback (Mazzoleni et al., 2015a). Further investigation revealed that the self-inhibition of growth was not limited to plant species, and was also observed to function in other organisms including bacteria, fungi, algae, protozoa, and insects in a concentration-dependent manner (Mazzoleni et al., 2015b). Such a profound discovery led plant scientists to identify the danger signals or DAMPs released from host tissues damaged/degraded by insect and microbial attacks (Duran-Flores and Heil, 2014; Barbero et al., 2016). A recent mechanistic and cell biology study revealed that non-self-eDNA penetrates root cells, leading to limited cell permeability, chloroplast dysfunction, and reactive oxygen species (ROS) generation, while self-eDNA maintains the intercellular space and triggers hypersensitive response and systemic acquired resistance (Chiusano et al., 2021). However, most of the previous studies focused on self-eDNA-induced plant autotoxicity rather than on plant immunity activation.

Recently, the application of self-eDNA in plants revealed the existence of self-eDNA-triggered immunity against microbial pathogens. Self-eDNA fragments shorter than 700 bp in size play a critical role in the indirect activation of plant immune system against bacterial pathogens through hydrogen peroxide (H_2_O_2_) and mitogen-activated plant kinase (MAPK) signaling (Duran-Flores and Heil, 2018).

Like DAMP signaling in animals, infiltration of *Arabidopsis thaliana* seedling leaves with single-stranded oligodeoxynucleotides (ssODNs) elicited defense response against *Pseudomonas syringae* pv. *tomato* (*Pto*) and *Botrytis cinerea* but not against *Tobacco mosaic virus* and inhibited growth via the BAK co-receptor and ROS generation (Toum et al., 2020). Besides plant self-eDNAs, a mixture of fragmented 100 μg/ml non-self-eDNAs derived from plant pathogenic fungi including *Phytophthora capsici, Fusarium oxysporum,* and *Rhizoctonia solani* reduced the mortality of pepper (*Capsicum annuum* L.) plants by up to 40% (Serrano-Jamaica et al., 2020). Transcriptome analysis of tomato (*Solanum lycopersicum* L.) leaves treated with fragmented self-eDNA revealed the induction of plant immune-related genes including pathogenesis-related (*PR*) proteins, calcium-dependent protein kinase 1 (*CPK1*), heat shock transcription factors (*HSFs*), heat shock proteins (*HSFs*), receptor-like kinases (*RLKs*), and ethylene-responsive factors (*ERFs*; Barbero et al., 2021). Although self-eDNA-induced plant immunity has been studied extensively, the topic of extracellular RNA (eRNA)-induced plant immunity has not been intensively exploited, with the exception of *Arabidopsis* leaf infiltration with a non-self-eRNA (bacterial rRNA), which activated plant resistance against *Pseudomonas syringae* pv. tomato (Lee et al., 2016). Nonetheless, the role of self-eRNA in plant immunity remains largely unknown.

In line with our previous discovery of bacterial eRNA as a trigger of plant immunity, we evaluated whether self-eRNAs activate resistance against microbial pathogens in pepper plants. Experiments were conducted under field conditions using pepper plants challenged with eRNAs derived from pepper (self-eRNA) and *Nicotiana benthamiana* (non-self-plant-eRNA), and those derived from virulent and avirulent pathogens. Considering the growth penalty caused by self-eDNA application, we measured plant growth and yield at the end of the growing season. Intriguingly, self-eRNA-induced immunity was enough to protect pepper plants against the naturally occurring *Tomato spotted wilt virus (TSWV)*. To the best of our knowledge, this is the first report of self-eRNA-induced plant immunity against microbial pathogens under field conditions.

## Materials and methods

### Preparation of seedlings and bacteria

Pepper (*Capsicum annuum* L. cv. Bulkala) and *Nicotiana benthamiana* (*Nb*) seeds were sown on autoclaved soil-less potting medium (Punong Horticulture Nursery Medium Low; Punong Co. Ltd., Gyeongju, South Korea) containing zeolite, perlite, color dust, and lime (pH = 4.5 to 7.5). The seedlings of both plant species were cultivated for 6 weeks at 28°C under 12 h light/12 h dark cycle and approximately 7,000 lux light intensity using fluorescent lamps. Leaves of 6-week-old plants were harvested, immediately frozen in liquid nitrogen, and stored at −80°C until needed for RNA extraction.

*Xanthomonas axonopodis* pv. *vesicatoria* (*Xav*) and *Pseudomonas syringae* pv. *tomato* (*Pto*) were cultured at 30°C for 48 h in plates containing Luria-Bertani (LB; Difco Laboratories, Detroit, MI, United States) agar and King’s B (KB; Difco Laboratories) agar media, respectively. Cultures of *Xav* and *Pto* were centrifuged at 16,000 × *g* for 5 min. The supernatant was discarded and the pellet containing bacterial cells was used to perform subsequent experiments.

### Preparation of plant and bacterial eRNAs

Total eRNAs were isolated from pepper cultivars Bulkala (for 2020 field trial) and Asia Jumbo (for 2021 field trial), *Nb* plants, and bacterial pathogens (*Xav* and *Pto*) using TRIzol Reagent (Invitrogen, Carlsbad, CA, United States), according to the standard protocol described previously (Lee et al., 2016).

To isolate bacterial eRNAs, the centrifuged *Xav* and *Pto* cells were mixed with TRIzol Reagent and incubated at room temperature (RT) for 5 min. Then, chloroform was added to each sample, and centrifugation was performed at 10,000 × *g* for 15 min at 4°C. Subsequently, the upper phase was transferred to a new tube, and nucleic acid was precipitated by adding isopropyl alcohol. The resultant bacterial RNA pellet was resuspended in nuclease-free water.

To isolate plant eRNAs, the previously frozen leaves of 6-week-old pepper and *Nb* seedlings were ground into a fine powder using a mortar and pestle. The powdered leaf tissue (100 mg) of each plant species was mixed with 1 ml of TRIzol Reagent and incubated at RT for 5 min. Plant eRNAs were isolated as described above.

The obtained bacterial and plant eRNAs were treated with RNase and diluted to a concentration of 100 ng/μl.

### Field trials

Field trials were conducted in April to August and 2021 in Nonsan, Chungcheongnam-do, South Korea (36.23577°N, 127.18946°E), where plants are occured by multiple viral diseases each year. All necessary permits were obtained from landowners to conduct these trials. Pepper seedlings were transplanted at a distance of 30 cm in furrows covered with black polyethylene film to prevent weed growth before transplanting. To test the induction of resistance under field conditions, leaves of 1-month-old pepper seedlings were infiltrated with 1 ml of 100 ng/μl plant and bacterial eRNAs. Leaves infiltrated with 1 ml of sterilized water were used as a negative control. Seedlings drenched with 1 mM benzothiadiazole (BTH; Bion 50 WG, Syngenta, Basel, Switzerland) served as a positive control. Plants roots were drenched with 50 mL of 1 mM BTH twice: first at 1 week prior to self-eRNA and non-self-eRNA infiltration, and again on the day of self-eRNA and non-self-eRNA infiltration. Each treatment was replicated four times in a completely randomized block design, with 12 plants per block in 2020 and 9 plants per block in 2021.

### Bacterial Pathogen Inoculation

Plants were challenged with *Xav* as described previously (Lee et al., 2017). Briefly, at one week after self-eRNA and non-self-eRNA (*Xav*, *Pto,* and *Nb*) infiltration (which coincided with 2 -3 weeks after field transplantation), two leaves per pepper seedling were infiltrated with 500 μL of *Xav* suspension (optical density (OD)_600_ = 0.01); overall, five plants per block in 2020 and six plants per block in 2021 were inoculated with *Xav*. One week after pathogen inoculation, disease severity on pepper leaves was scored on a scale of 0–5, as follows: 0, no symptom; 1, mild chlorosis; 2, chlorosis; 3, severe chlorosis and mild necrosis; 4, necrosis; 5, necrosis with cell death.

### Diagnosis of naturally occurring viral diseases

To evaluate virus titers in field-grown plants, qRT-PCR was performed as described previously (Kong et al., 2018). Briefly, ten leaves per replication were randomly sampled 90 days after plant and bacterial eRNA infiltration and BTH application, and immediately frozen in liquid nitrogen. Total eRNA was isolated from the frozen leaves using TRIzol Reagent (Molecular Research Inc., Cincinnati, OH, United States), according to the manufacturer’s instructions and as described in our previous study (Lee et al., 2017; Kong et al., 2018). First-strand cDNA was synthesized from 2 μg of DNase-treated total eRNA using oligo dT primers and Moloney murine leukemia virus reverse transcriptase (Enzynomics, Daejeon, Korea). Then, qRT-PCR was performed using the synthesized cDNA, iQ™ SYBR® Green Supermix (Bio-Rad Inc., Hercules, CA, United States), and 10 pM primers under the following cycling conditions: initial polymerase activation for 10 min at 95°C, followed by 40 cycles of 30 s at 95°C, 60 s at 55°C, and 30 s at 72°C. Viral sequence-specific primer pairs were used to identify *Tomato yellow leaf curl virus* (*TYLCV; TYLCV*-F: 5′-CGCCCGCCTCGAAGGTTC-3′; *TYLCV*-R: 5′-TCGTCGCTTGTTTGTGCCTTG-3′) and *TSWV* (*TSWV*-F: 5′-ATGTCTAAGGTTAAGCTCAC-3′; *TSWV*-R: 5′-TCAAGCAAGTTCTGCGAGTT-3′), as described previously (Kong et al., 2018). Gene transcript levels were normalized relative to that of the pepper *ubiquitin* (*CaUBQ*) gene, which was amplified using primers *CaUBQ*-F (5′-GCACAAGCACAAGAAGGTTAAG-3′) and *CaUBQ*-R (5′-GCACCACACTCAGCATTAGGA-3′). Relative transcript levels were calculated using the 2^–ΔΔCT^ method. Standard error of means among replicates were calculated using JMP IN ver. 4.0 (SAS Institute Inc., Cary, NC, United States) and Bio-Rad manager ver. 2.1 (Bio-Rad CFX Connect).

### Expression Analysis of Defense-related genes

The expression of defense-related genes, including *Defensin (CaDEF), Chitinase type 2 (CaCHI2), 9-Lipoxygenase (CaLOX1)*, and *Pathogenesis-related 4 (CaPR4)*, was evaluated in pepper plants at the end of the growing season (i.e., at 90 days after RNA leaf-infiltration and before harvesting) by qRT-PCR. Pepper cDNA was prepared as described above, and qRT-PCR was performed using gene-specific primer pairs listed in Supplementary Table S1 (Huh et al., 2015; Kong et al., 2018), according to the same protocol as that used for virus quantification (described above).

### Assessment of plant yield

To investigate whether plant and bacterial eRNAs influence plant growth, fruit number and weight per plant were recorded in plant and bacterial eRNA treatments and compared with the corresponding values obtained in water and BTH treatments. The commercially valued red pepper fruits were harvested twice from mid- to end-August, which coincided with approximately 100 days post-treatment. Fruit number per plant was recorded at each harvest. Total fruit weight per plant was also calculated at each harvest.

### Direct Effect of Pepper eRNA on *Xanthomonas axonopodis* pv. *vesicatoria’* Growth

*Xav* was cultured in LB agar at 30°C overnight. A single colony was then used to inoculate freshly prepared LB broth, and cultured in an incubator at 220 rpm and 30°C. 20 μl of pre-culture was added to 180 μl of fresh full-strength, 10-fold diluted (0.1), and 100-fold diluted (0.01) LB broth into a 96-well plate. To inoculate each medium with the same number of *Xav* cells, the bacterial cells were spun down by centrifugation at 8,000 rpm for 5 min at room temperature. The bacterial cell pellets were resuspended in full-strength, 10-fold dilution, and 100-fold dilution treatments, and then transferred into a 96-well plate. Then, 100 ng/μl pepper self-eRNA and *Pto* non-self-eRNA were used to inoculate each medium, which was pre-inoculated with *Xav* at (OD_600_ = 0.2). Polymyxin B (32 μg/ml), an antibiotic that kills Gram-negative bacteria, was used as a positive control. Treatment with *Xav* alone was used as a negative control. The growth of *Xav* was monitored for 45 h using Spark™ 10M multimode microplate reader (Tecan Trading AG, Switzerland).

### Statistical analysis

The experimental datasets were subjected to ANOVA using JMP IN software. The statistical significance of differences among treatments was determined based on the *F*-value at *p* = 0.05. When a significant F-value was obtained for treatments, separation of means was accomplished using Fisher’s protected least significant difference (LSD) test at *p* = 0.05.

## Results

### Self- and non-self-eRNAs induce plant immunity under field conditions

**Figure 1:**
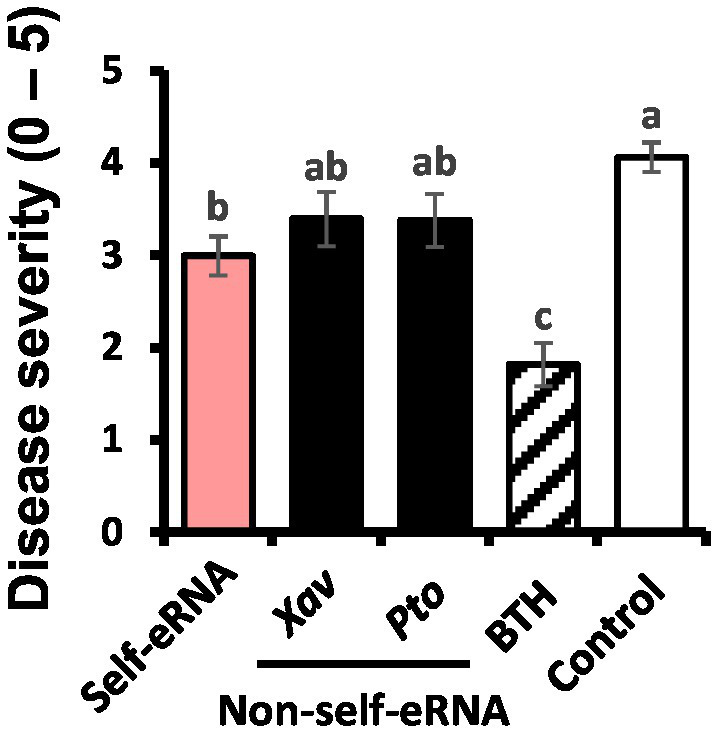
Activation of plant immunity in pepper plants treated with extracellular RNAs (eRNAs) and benzothiadiazole (BTH) under field conditions. Leaves of pepper plants were infiltrated with self-eRNA (derived from pepper plants) and non-self-bacterial eRNAs (OD_600_ = 0.01; derived from virulent pathogens, *Xanthomonas axonopodis* pv. *vesicatoria* [*Xav*] and *Pseudomonas syringae* pv. *tomato* [*Pto*]), and disease severity was determined at 7 days post-infiltration. Data represent mean ± standard error of mean (SEM). Different letters indicate statistically significant differences between eRNA/BTH and water (control) treatments (*p* = 0.05; least significant difference [LSD] test).

The severity of bacterial spot disease on pepper seedlings treated with self-eRNA was 2.99, which was 1.4-fold lower than that on plants treated with water (negative control; disease severity = 4.06; Figure 1). Plants treated with non-self-eRNAs, including *Xav* and *Pto* eRNAs, showed no significant difference in disease severity compared with the control. However, plants treated with BTH exhibited a 2.2-fold reduction in disease severity compared with the control.

**Figure 2:**
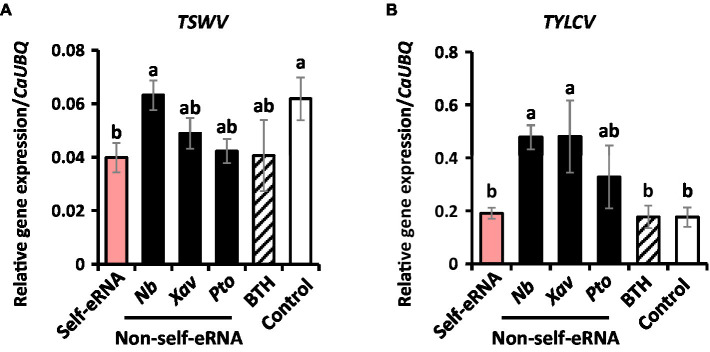
Self-eRNA treatment induces plant immunity against the naturally occurring *Tomato spotted wilt virus (TSWV)* and *Tomato yellow leaf curl virus (TYLCV)*. Induction of immunity against *TSWV* **(A)** and *TYLCV* **(B)** in pepper plants by pre-infiltration with self-eRNA (derived from pepper) and non-self-eRNA derived from *N. benthamiana (Nb)*, *X. axonopodis pv. vesicatoria (Xav)*, and *P. syringae pv. tomato (Pto)*. BTH treatment used as a positive control. The expression of immunity-related genes was evaluated by qRT-PCR at 90 days post-infiltration. The housekeeping gene *CaUBQ* was used as an internal reference. Data represent mean ± SEM. Different letters indicate statistically significant differences between control and other treatments (*p* = 0.05; LSD test).

In control plants, the titer of naturally occurring *TSWV* and *TYLCV* was 0.062 and 0.18, respectively, as shown by qRT-PCR analysis. The titer of *TSWV* in self-eRNA-treated plants was 0.04, which was 1.6-fold lower than that in control plants (Figure 2A). Non-self-eRNA treatments did not show any difference relative to the control (Figure 2A). The titer of *TYLCV* showed no significant difference among treatments (Figure 2B).

### Measurement of pepper fruit yield

**Figure 3:**
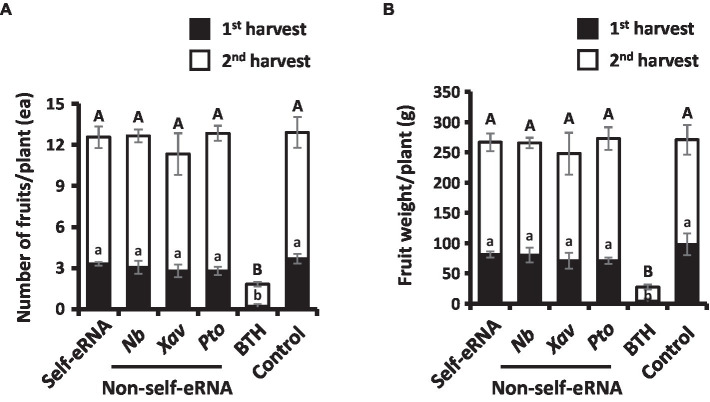
Effect of self- and non-self-eRNAs on pepper marketable yield. a, b Fruit number **(A)** and fruit weight **(B)** per plant treated with self-eRNA derived pepper and non-self-eRNA that including *N. benthamiana (Nb)*, *X. axonopodis pv. vesicatoria (Xav)*, and *P. syringae pv. tomato (Pto)*, 1 mM BTH, and control. Fruit number and weight were measured in the second round of harvest at 80 and 90 days post-infiltration, respectively. Data represent mean ± SEM. Different letters indicate statistically significant differences between control and other treatments (*p* = 0.05; LSD test).

Fruit number and weight showed no significant difference among the various treatments, except the BTH treatment (Figures 3A,B). The number and weight of fruits in BTH-treated plants were 12.91 per plant and 270.83 g, respectively, indicating a reduction by 7.12- and 10.00-fold compared with the control (Figures 3A,B).

### Upregulation of Defense-related Genes by Self-eRNA Treatment

**Figure 4:**
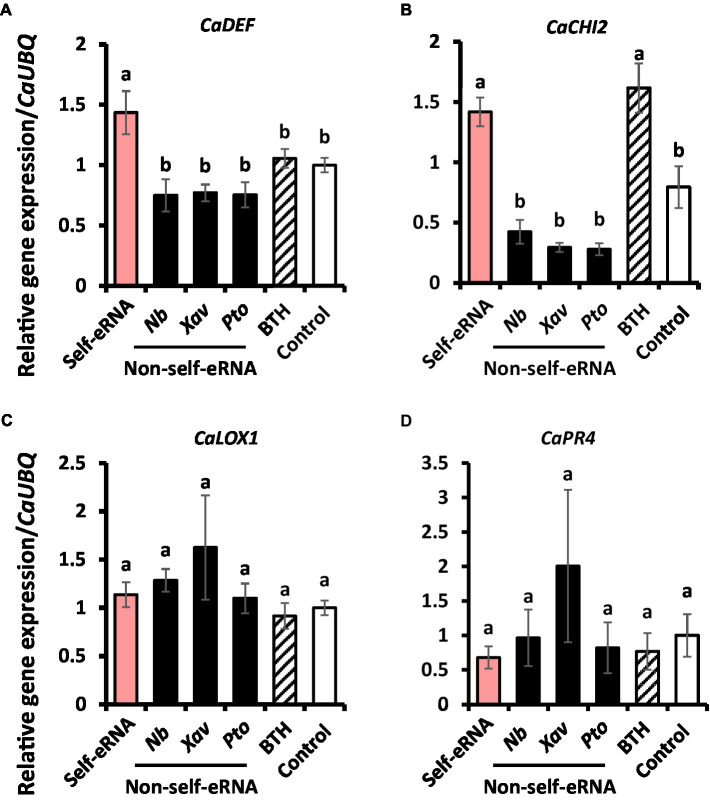
Induction of defense-related genes in pepper following self-eRNA treatment under field conditions. Quantifications of the expression of marker genes of the *CaDEF* **(A)**, *CaCHI2* **(B)**, *CaLOX1* **(C)**, and *CaPR4* **(D)**. *CaDEF* related abscisic acid, salicylic acid, and jasmonic acid signaling pathways in plants. *CaCHI2* related abscisic acid, ethylene, and jasmonic acid signaling pathways in plants. *CaLOX1* related salicylic acid and ethylene signaling pathways in plants. *CaPR4* related jasmonic acid signaling pathway in plants. Plants treated with self-eRNA and non-self-eRNA that including *N. benthamiana* (*Nb*), *X. axonopodis* pv. *vesicatoria* (*Xav*), and *P. syringae* pv. tomato (*Pto*), 1 mM BTH, and control. *CaUBQ* was used as a housekeeping gene for data normalization. Data represent mean ± SEM. Different letters indicate statistically significant differences between control and other treatments (*p* = 0.05; LSD test).

To investigate the mechanism of immune response activation in pepper by self-eRNA treatment, the expression of defense-related genes was examined. Relative expression levels of *CaDEF* and *CaCHI2* in self-eRNA treated plants were increased by 1.43- and 1.42-fold, respectively, compared with the control (Figures 4A,B), while those of *CaLOX1* and *CaPR4* showed no significant differences relative to the control (Figures 4C,D). Both *CaDEF* and *CaCHI2* are related to ABA and JA signaling (Hong et al., 2000; Hong and Hwang, 2002; Do et al., 2004), whereas *CaPR4* and *CaLOX1* are known as SA and JA marker genes (Hwang and Hwang, 2009; Hwang et al., 2014). Together, these findings suggest that self-eRNA elicited pepper immunity through ABA signaling.

### Direct Effect of Self-eRNA on *Xanthomonas axonopodis* pv. *vesicatoria’* Growth

**Figure 5:**
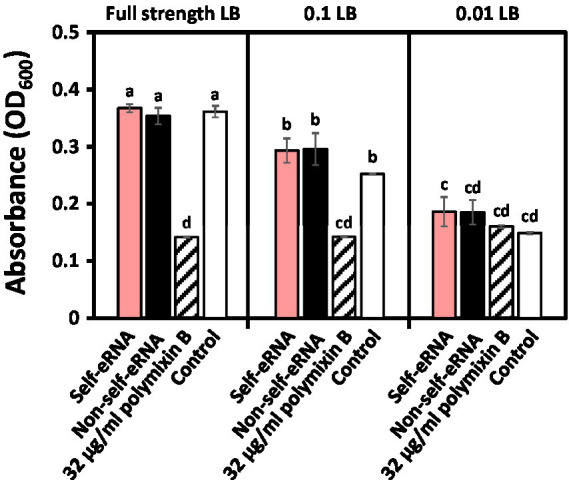
Evaluation of the direct effect of self-eRNA treatment on the growth of *X. axonopodis* pv. *vesicatoria (Xav).* Growth of *XaV* amended with 100 ng/μl self-eRNA (pepper) and non-self-eRNA (*P. syringae* pv. *tomato*) was monitored in full-strength LB (left panel), 10-fold diluted (0.1) LB (middle panel), and 100-fold diluted (0.01) LB (right panel) for 45 h. Polymyxin B (32 μg/ml) was used as a positive control. Data represent mean ± SEM (*n* = 3). Different letters indicate statistically significant differences between control and other treatments (*p* = 0.05; LSD test).

To evaluate the direct effect of self-eRNA on the growth of *Xav*, systemic translocation of the introduced self-eRNA was monitored. The OD600 of *Xav* amended with 100 ng/μl self-eRNA and non-self-eRNA (*Pto*) was 0.37 and 0.35 respectively, while that of the polymyxin B treatment was 0.14 in full-strength LB (Figure 5 left panel). Bacterial growth in all treatments, except polymyxin B treatment, showed no significant difference compared with the control (Figure 5 left panel). In 10-fold diluted LB, the OD_600_ of self-eRNA and non-self-eRNA was 0.29 and 0.30, respectively (Figure 5 middle panel). In 100-fold diluted LB, no statistically significant differences were detected among the various treatments (Figure 5 right panel). These results indicate that self-eRNA and non-self-eRNA could not directly inhibit the growth of *Xav*.

## Discussion

Our results confirmed the activation of plant systemic immunity by self-eRNA in field-grown pepper plants. Plant perception of nucleic acids (e.g., RNA and DNA) had not been intensively studied until 2015 (Mazzoleni et al., 2015a). In the field, self-eRNA clearly activated plant immunity against bacterial and viral pathogens (Figures 1, 2). The systemically translocated eRNA itself did not alter the bacterial pathogen growth (Figure 5) indicating that plants were protected by the activation of systemic immunity.

Compared with the autotoxicity induced in plants by self-eDNA application previously (Barbero et al., 2016; Duran-Flores and Heil, 2018; Serrano-Jamaica et al., 2020; Toum et al., 2020), it is noteworthy that self-eRNA-induced plant immunity was not accompanied by any growth penalty (also referred to as the allocation fitness cost; Figure 3). We cannot explain why allocation fitness cost is not required in the field trial of self-eRNA, but we have two hypotheses. Firstly, we hypothesize that self-eRNA-elicited plant immunity is not dependent on ROS and SA-mediated PR protein activation, which are strongly induced by self-eDNA treatment (Duran-Flores & Heil, 2018; Chiusano et al., 2021). Previous studies demonstrated that ROS and PR protein activation are related to the SA-dependent signaling pathway and result in a strong growth penalty (Clarke et al., 2000; Heil et al., 2000; Noutoshi et al., 2005; Walters and Heil, 2007; Kouzai et al., 2018). In the current study and many previous studies, the application of BTH (also known as acibenzolar-S-methyl) significantly reduced plant growth (Clarke et al., 2000; Heil et al., 2000; Noutoshi et al., 2005; Walters and Heil, 2007; Kouzai et al., 2018). The mode of action of BTH (as an SA analog) on the allocation fitness cost and plant immunity induction demonstrates the induction of ROS and PR proteins (Miura et al., 2013; Huot et al., 2014; Herrera-Vásquez et al., 2015; Kouzai et al., 2018; Poór, 2020). Secondly, we speculated that defense signaling can be activated independent of SA signaling, such as induced systemic resistance, by plant growth-promoting rhizobacteria (PGPR). Previous studies demonstrated that the application of PGPR and bacterial determinants on pepper and other plant species at the seedling stage successfully protected them from bacterial and viral pathogens and had no detrimental effect on plant growth; rather, plant growth was enhanced in many cases (Kong et al., 2018). Characterization of PGPR-mediated induced systemic resistance is mostly dependent on jasmonic acid (JA) and ethylene signaling rather than on SA signaling, which is activated by necrotizing pathogen-induced systemic acquired resistance (Van Der Ent et al., 2009; Beneduzi et al., 2012). Sufficient data are not yet available to determine whether JA and ethylene signaling mediate self-eRNA-induced plant immunity. Detailed mechanistic analysis of defense signaling by self-eRNA treatment under controlled conditions will enable us to understand the relationship between plant immunity and growth.

Some other questions, such as how plants recognize self-eRNA and which epitope of self-eRNA is perceived by plant receptors, remain unanswered. Despite RNA-sequencing and *Arabidopsis* mutant analysis, plant receptors potentially involved in the perception of non-self-eRNA have not been identified to date (Lee et al., 2016). However, in animal cells, the mechanism of self-eRNA perception has been proposed and confirmed (Schlee and Hartmann, 2016; Heil and Vega-Muñoz, 2019). For example, in the mammalian system, Toll-like receptors (3, 7, 8, and 10), RIG-like receptors, and protein kinase R (PKR) have been reported to function as mammalian RNA sensors (Alexopoulou et al., 2001; Kato et al., 2006; Mancuso et al., 2009; Mayo and Cole, 2017; Heil and Vega-Muñoz, 2019). Further molecular and biochemical evaluation is needed to identify potential plant receptor(s) using *Arabidopsis* as a model plant species. It would be interesting to determine the eRNA epitope that directly binds to a plant receptor. Similar to the case study of non-self-eRNA in *Arabidopsis*, differential fractionation of eRNA could be conducted in pepper to identify the eRNA determinant by screening for the activation of plant immunity and the biochemical response, such as callose deposition, to microbe-associated molecular pattern (MAMP) recognition by self-eRNA (Lee et al., 2016). Based on the previous studies of pattern recognition, rRNA, tRNA, small RNA, and each fragmented product are good candidates for MAMPs. The self-eRNA, as an example of DAMP and MAMP, must be conserved within and variable between plant species.

ABA signaling is widely regarded as an important player in plant immunity as well as in critical abiotic stress responses (Lievens et al., 2017). ABA signaling is involved in plant defense against insect pests including thrips, which is a well-known vector of plant DNA viruses (Geminivirus) such as *TSWV* (Bedford et al., 1994; Escobar-Bravo et al., 2018; Guo et al., 2020). We speculate that the lower level of TSWV in self-eRNA-pretreated pepper plants is the result of reduced virus-vector infestation through plant immune activation *via* ABA signaling (Figures 2, 4A,B). Moreover, the long-lasting immune memory conferred by self-eRNA suggests its potential for field applications.

In conclusion, we report, for the first time, self-eRNA-induced plant immunity against bacterial and viral pathogens in pepper. Our results were obtained from field trials, indicating that self-eRNA can potentially be applied to plants in the agricultural field in the near future. Moreover, self-eRNA-induced immune activation is advantageous, since it is not compromised by a growth penalty. However, the concept of self-eRNA-induced plant immunity is in its infancy, and intensive investigation is required to understand why and how plants manipulate the balance between immunity and growth.

## Data availability statement

The original contributions presented in the study are included in the article/Supplementary material, further inquiries can be directed to the corresponding author.

## Author contributions

DK conducted the field trial, performed viral diagnosis. MR examined the expression of defense-related genes and participated in the field trial. DK and MR created the figures and wrote the manuscript. S-KO reviewed the manuscript. C-MR conceived the study, participated in its design and coordination, and wrote the manuscript.

## Funding

This research was supported by grants from the Rural Development Administration (RDA), Strategic Initiative for Microbiomes in Agriculture and Food, Ministry of Agriculture, Food and Rural Affairs, Republic of Korea (as part of the multi-ministerial Genome Technology to Business Translation Program; 918017-4), Center for Agricultural Microorganism and Enzyme (Project No. PJ015049) of RDA, and the KRIBB Initiative Program, South Korea.

## Conflict of interest

DK, MR, and CM-R were employed by the company KRIBB.

The remaining author declares that the research was conducted in the absence of any commercial or financial relationships that could be construed as a potential conflict of interest.

## Publisher’s note

All claims expressed in this article are solely those of the authors and do not necessarily represent those of their affiliated organizations, or those of the publisher, the editors and the reviewers. Any product that may be evaluated in this article, or claim that may be made by its manufacturer, is not guaranteed or endorsed by the publisher.
