# Supplementary material for: Extracellular self-RNA: A danger elicitor in pepper induces immunity against bacterial and viral pathogens in the field
Source: Front Plant Sci. 2022 Sep 26;13:864086. doi: 10.3389/fpls.2022.864086 (PMC9549290; doi:10.3389/fpls.2022.864086)
Supplement: Supplementary file 1 [file Table_1.DOCX]

**Supporting Information**

**Title:** Extracellular self-RNA induces immunity against bacterial and viral pathogens in pepper under field condition

Doyeon Kim^1,2†^, Myoungjoo Riu^1,3†^, Sang-Keun Oh^3,*^, and Choong-Min Ryu^1,2,*^

^1^Molecular Phytobacteriology Laboratory, Infectious Disease Research Center, KRIBB, Daejeon 34141, South Korea

^2^Department of Biosystems and Bioengineering, KRIBB School of Biotechnology, University of Science and Technology, Daejeon 34113, South Korea

^3^Department of Applied Biology, College of Agriculture & Life Sciences, Chungnam National University, Daejeon 34134, South Korea

^†^These authors contributed equally to this work

***** **Corresponding authors:**Sang-Keun Oh
sangkeun@cnu.ac.kr

Choong-Min Ryu
cmryu@kribb.re.kr

The following Supporting Information is available for this article:

**Table S1** **Primers used in this study**

| **Primer** | **Sequence (5’ to 3’)** |
| --- | --- |
| TYLCV-F | CGCCCGCCTCGAAGGTTC |
| TYLCV-R | TCGTCGCTTGTTTGTGCCTTG |
| TSWV-F | ATGTCTAAGGTTAAGCTCAC |
| TSWV-R | TCAAGCAAGTTCTGCGAGTT |
| CaUBQ-F | GCACAAGCACAAGAAGGTTAAG |
| CaUBQ-R | GCACCACACTCAGCATTAGGA |
| CaDEF1-F | CACACTCCATGCGTTTCTTT |
| CaDEF1-R | GTTCTTGCCTCAACAATTCTCA |
| CaCHI2-F | ATTGGACGATGGAAGCCATCACCAG |
| CaCHI2-R | ATATTCCGAATGTCTAAAGTGGTAC |
| CaLOX1-F | AATCCATTGTTCAGTTCCTTATCC |
| CaLOX1-R | CCTCCTTCATAAAGCCTCAGT |
| CaPR4-F | GAACACAAGCAACGGTGAGA |
| CaPR4-R | GGCACTTGTTTAGGCAGAGC |
