# Supplementary figures and images for: Extracellular self-RNA: A danger elicitor in pepper induces immunity against bacterial and viral pathogens in the field
Source: Front Plant Sci. 2022 Sep 26;13:864086. doi: 10.3389/fpls.2022.864086 (PMC9549290; doi:10.3389/fpls.2022.864086)

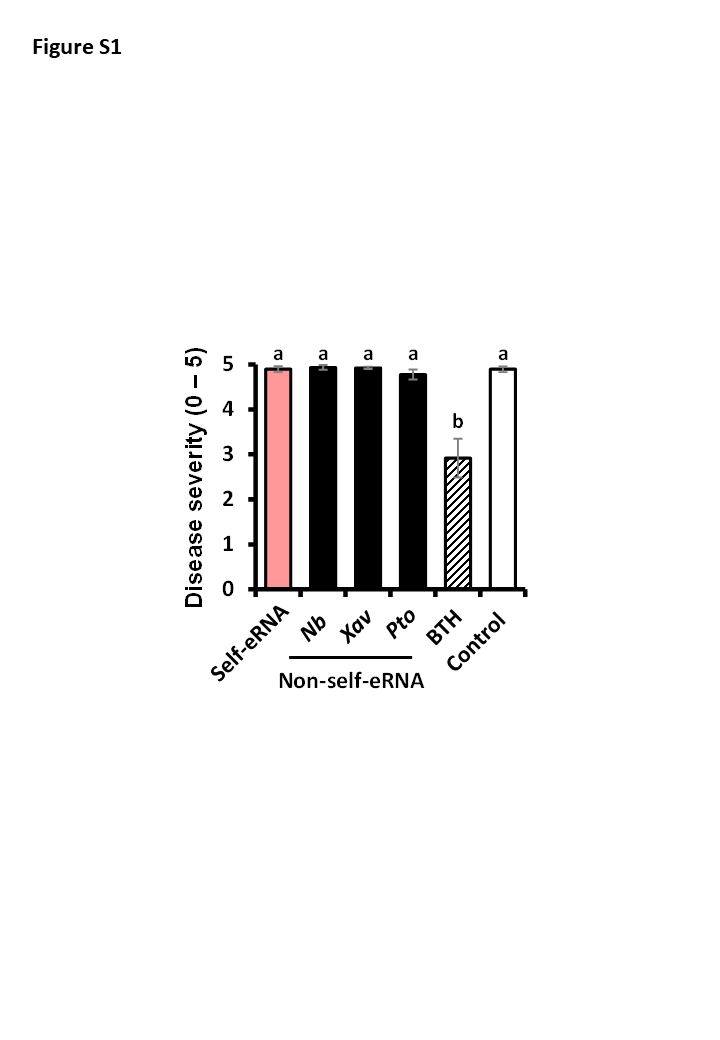

Supplement: Supplementary file 2 [file Image_1.TIF]

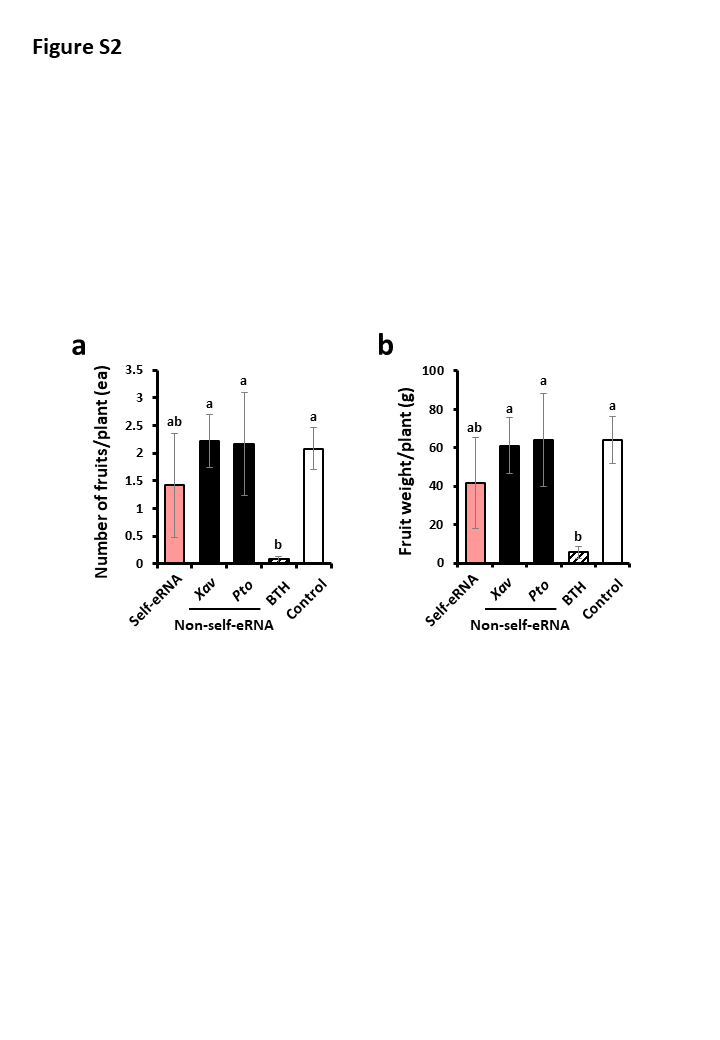

Supplement: Supplementary file 3 [file Image_2.TIF]

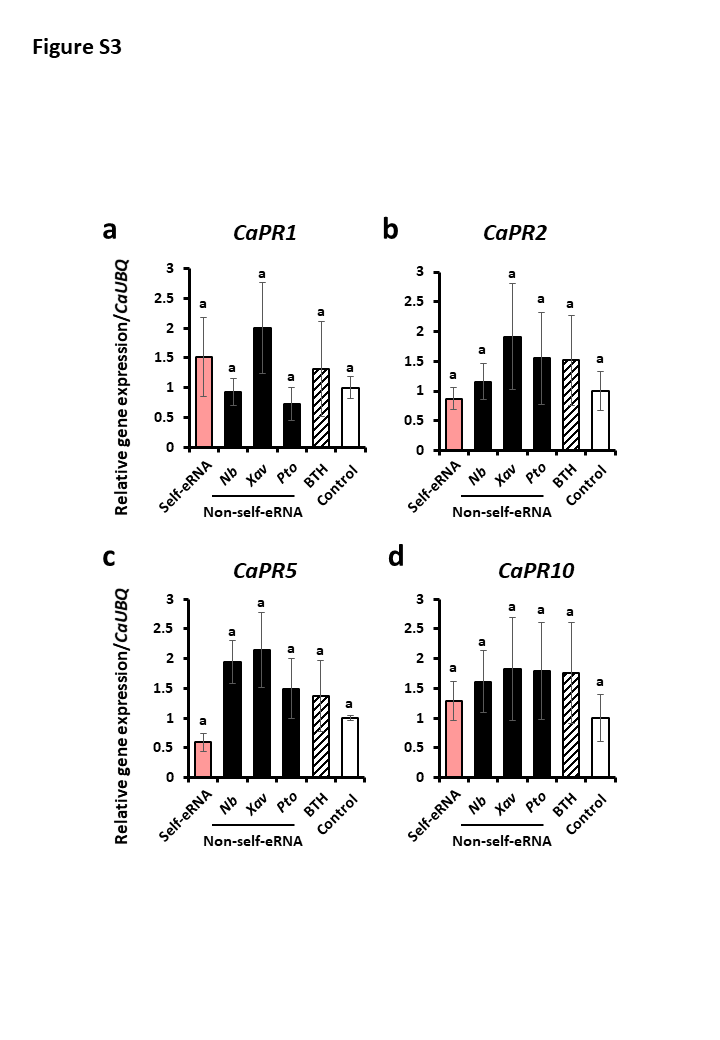

Supplement: Supplementary file 4 [file Image_3.TIF]
